# Low Computational Coding-Efficient Distributed Video Coding: Adding a Decision Mode to Limit Channel Coding Load

**DOI:** 10.3390/e25020241

**Published:** 2023-01-28

**Authors:** Shahzad Khursheed, Nasreen Badruddin, Varun Jeoti, Dejan Vukobratovic, Manzoor Ahmed Hashmani

**Affiliations:** 1 Department of Electrical and Electronic Engineering, Institute of Health and Analytics, Universiti Teknologi PETRONAS, Seri Iskandar 32610, Malaysia; 2 Faculty of Technical Sciences, University of Novi Sad, 21000 Novi Sad, Serbia; 3 High Performance Cloud Computing Center (HPC3), Department of Computer and Information Sciences, Universiti Teknologi PETRONAS, Seri Iskandar 32610, Malaysia

**Keywords:** low complexity encoder DVC, low channel coding complexity, DRVC, coding efficient DVC, coding efficient DRVC

## Abstract

Distributed video coding (DVC) is based on distributed source coding (DSC) concepts in which video statistics are used partially or completely at the decoder rather than the encoder. The rate-distortion (RD) performance of distributed video codecs substantially lags the conventional predictive video coding. Several techniques and methods are employed in DVC to overcome this performance gap and achieve high coding efficiency while maintaining low encoder computational complexity. However, it is still challenging to achieve coding efficiency and limit the computational complexity of the encoding and decoding process. The deployment of distributed residual video coding (DRVC) improves coding efficiency, but significant enhancements are still required to reduce these gaps. This paper proposes the QUAntized Transform ResIdual Decision (QUATRID) scheme that improves the coding efficiency by deploying the Quantized Transform Decision Mode (QUAM) at the encoder. The proposed QUATRID scheme’s main contribution is a design and integration of a novel QUAM method into DRVC that effectively skips the zero quantized transform (QT) blocks, thus limiting the number of input bit planes to be channel encoded and consequently reducing both the channel encoding and decoding computational complexity. Moreover, an online correlation noise model (CNM) is specifically designed for the QUATRID scheme and implemented at its decoder. This online CNM improves the channel decoding process and contributes to the bit rate reduction. Finally, a methodology for the reconstruction of the residual frame ($\hat{R}$) is developed that utilizes the decision mode information passed by the encoder, decoded quantized bin, and transformed estimated residual frame. The Bjøntegaard delta analysis of experimental results shows that the QUATRID achieves better performance over the DISCOVER by attaining the PSNR between 0.06 dB and 0.32 dB and coding efficiency, which varies from 5.4 to 10.48 percent. In addition to this, results determine that for all types of motion videos, the proposed QUATRID scheme outperforms the DISCOVER in terms of reducing the number of input bit-planes to be channel encoded and the entire encoder’s computational complexity. The number of bit plane reduction exceeds 97%, while the entire Wyner-Ziv encoder and channel coding computational complexity reduce more than nine-fold and 34-fold, respectively.

## 1. Introduction

A distributed video coding (DVC) scheme is based on two important theorems, Slepian-Wolf [1] and Wyner-Ziv [2]. This video coding paradigm follows the principle of distributed source coding (DSC) and is becoming a prominent video coding paradigm due to shifting the high computational complexity to the decoder. Moreover, it came up as a promising scheme for computationally lightweight and limited resources applications [3,4] such as wireless video sensor networks, due to the independent encoding of video frames at the encoder and their joint decoding at the decoder [4]. However, poor rate-distortion (RD) performance and high delay due to feedback channels are major challenges in this video coding paradigm. The channel decoding needs a feedback channel for error correction and exhibits high computational complexity. Even today, the RD performance offered by DVC still lags the conventional motion-compensated predictive video codecs [4], such as H.264/AVC. One of the most adopted DVC frameworks in the literature is DISCOVER [5]. It outperforms the H.264/AVC intra-encoder RD performance for simple motion videos, while having worse performance for complex and high motion videos.

Different factors contribute to the coding performance gap between DVC and H.264/AVC, including the inferior performance of channel coding tools, the correlation noise model (CNM) inaccuracies, and inferior side information (SI) quality. Many techniques have been proposed to improve the RD performance in DVC at the expense of system complexity. Some of these techniques include the hash methods used at the encoder, encoder-based CNM estimation, encoder-based quality control, encoder-based motion estimation, etc. [6,7,8]. These methods increase the encoder’s complexity.

In DVC, SI has a significant impact on RD performance. This is because the high-quality SI leads to higher compression efficiency and lower bit rates. Since the decoder does not have access to the current Wyner-Ziv (WZ) frame, the hash code is generated at the cost of extra computation at the encoder. Usually, the cyclic redundancy check (CRC) [9] is generated as hash code and sent as auxiliary information to assist in the SI generation process [10,11,12,13,14,15,16,17,18]. Among these hash code-based encoders, some codecs presented encoder-based adaptive hash generation strategies to obtain the optimal RD performance. In addition, other computationally expensive information, such as entropy encoded most significant bit-plane [19,20] or intra-encoded down-sampled WZ frame [21], can be sent as hash information. In [17], the authors presented detailed analyses of hash-based motion estimation. Their experimental results depict that the hash-based strategy assists in achieving high quality SI for video sequences with medium to high bit rates (motion). However, this strategy is not beneficial for low motion videos. Further, it is suggested that SI quality is directly proportional to the number of hash codes. A large number of hash codes generate high quality SI and vice versa. This hash-based codec increases the encoder’s computational complexity by generating a large number of hash codes. The hash-based techniques did not focus on reducing the workload of the channel encoding process, which is a major element of the encoder’s computational complexity.

Quantization is another key technique used in the DVC encoder for rate control and coding efficiency. Due to its simplicity, scalar quantization is used in the majority of existing DVC schemes. Multiple DVC codecs have presented adaptive quantization techniques [22,23,24,25]. The authors in [24] explored the three distinct types of adaptive quantization methods: adaptive sub-band level quantization, adaptive frame-level quantization, and overall adaptive quantization [24]. The perceptual distortion probability for overall adaptive quantization is established first to determine the target perceptual distortion of SI. This technique is somewhat complicated since SI must be created at the encoder for perceptual distortion probability estimation. The best quantization matrix is identified adaptively and iteratively. The SI quality, RD optimization, and perceptual features are combined with the estimated SI and target perceptual distortions for quantization matrix identification. The authors in [23] propose a complicated encoder-based optimal entropy-constrained non-uniform scalar quantizer for pixel domain DVC (PDDVC). First, the encoder employs a conditional probability density function for the estimation of the rate and distortion model. Then, an optimization function for RD is developed. A modified Lloyd-Max technique with a novel quantization partition updating approach is applied to optimize the RD function. Experimental findings of [23,24] indicate that suggested quantization techniques enhance RD performance. However, these established algorithms increased the encoder’s computational complexity. Furthermore, no progress has been made to reduce the input to channel encoding to decrease its computational complexity.

Techniques other than adaptive quantization and hash methods are deployed at the encoder to increase the coding efficiency. The literature [26] provided an encoder-based SI interpolation approach for obtaining the global motion vector by using the SI frame interpolation algorithm. The suggested approach is capable of improving the quality of SI, however, at the cost of increased encoder complexity due to the feature-point matching process deployed at the encoder. The research work [27] presented the Human Visual system (HVS) based DVC technique. Due to its underlying temporal and spatial sensitivity and masking properties, HVS is rarely able to detect the changes below the just noticeable difference (JND) distortion threshold. Therefore, correcting the unnoticeable signal difference between the original frame and SI is unnecessary. To deploy the JND model at encoder basic SI is generated. The basic SI is required to be generated at the encoder to deploy the JND model. Experimental findings indicate that bit rate is reduced significantly with the proposed model at the expense of the SI generation and JND calculation complexity.

In any of the DVC codecs, one of their major components is the channel coding process utilized for error correction. The iterative decoding process, associated with the error correction, is a time consuming task that increases the complexity of a WZ decoder. Currently, the low-density parity-check accumulate (LDPCA) codes [28] are a popular choice for Wyner-Ziv (WZ) video coding and are considered to exhibit lower complexity compared to previous channel coding techniques. However, their complexity is still dominant in the overall DVC decoding process.

The most challenging issue in DVC is a trade-off between the RD performance and the encoder complexity and the channel coding process. This research presents the encoder-based scheme for distributed residual video coding (DRVC) that limits the number of input bit planes passed through the channel coding process, thus maintaining the low encoder complexity, and improving the coding efficiency. The DRVC codec with proposed attributes is named QUAntized Transform ResIdual Decision (QUATRID). The primary attributes of this research work include the following:

1. We propose a QUantized TrAnsform Decision Mode (QUAM) for DRVC that drops the zero QT blocks of the residual frame. QUAM generates fewer bit planes to be channel encoded, thus reducing the complexity of the channel encoding. Similarly, the fewer channel-encoded bit planes reduce the complexity of channel decoding. Therefore, QUAM reduces the overall computational complexity while improving coding efficiency.
2. We propose and deploy the online correlation noise model (CNM) at the decoder to perform the error correction of the bit planes generated using a limited number of nonzero QT blocks. The decision mode information (DMI) is utilised to form CNM.
3. We introduce the algorithm for the final reconstructed WZ frame reconstruction. The algorithm combines the blocks taken from SI based on DMI provided by the encoder and decoded quantized blocks. The primary contribution is designing an algorithm for reconstructing residual frames from a set of combined SI blocks and decoded quantized blocks.

The rest of this work is organized in the following manner: Section 2 discusses the related research studies that have been conducted on DRVC. Section 3 presents the proposed QUATRID codec in detail. The experimental findings are presented and discussed in Section 4. Finally, the research findings are concluded in Section 5.

## 2. Related Work of DRVC and DVC

The DVC architecture is mainly classified into Transform-Domain DVC (TDDVC) [29], Pixel-Domain DVC (PDDVC) [30], and Distributed residual video coding (DRVC) [31]. The authors of [31] focus on the DRVC by computing and coding the residual frames in the pixel domain (PD). Similar to other DVC codecs, in DRVC, the video sequence is arranged into keyframes (KF) and Wyner-Ziv frames (W). The residual frame (R) for any W is computed simply by $R=W-{\;W}_{\mathrm{re}}$, where $W_{\mathrm{re}}$ is a simple estimation of the frame W. Usually, $W_{\mathrm{re}}$ is estimated by averaging the previous and next frames of the current W frame. The authors further considered that $W_{\mathrm{re}}$ is available to both the encoder and the decoder. First, the R frame is quantized and channel encoded. Then only parity bits are saved and transmitted. At the decoder, the side information (Y), the replica of W, is computed. Then $R^{\prime }=Y-{\;W}_{\mathrm{re}}$ is computed and decoded with Wyner-Ziv decoding. Finally, the Wyner-Ziv frame W is reconstructed. In PD, all the quantized values are required to be encoded. Therefore, computational complexity at the encoder increases as all these values are channel encoded.

In research works [32,33], the frame-level encoder rate control (ERC) and encoder block mode decision (EBMD) are proposed for the DRVC. This proposed EBMD depends on the individual residual pixels instead of measuring the block difference, distortion function, or compression rate. The experimental findings show that codec RD performance is better for low motion videos. In [4], the same authors proposed the three levels dead zone quantizer for PD-based DRVC. This quantizer maps large values set into three levels. Mostly, it maps low level input values to zero. In addition to this, the bit plane block-based (BPBB) scheme and bit plane re-arrangement (BPRA) method are presented. The three levels quantizer generated the two types of bit planes. The BPBB arranged each bit plane into 4 × 4 blocks and classified them into 1-block and 0-block. These two classified bit planes (1-block and 0-block) are fed for channel encoding. The BPRA removes the bits from the quantizer generated bit planes. It requires two different channel encoders and shows the mixed RD performance for different video sequences.

The two different DRVC schemes are described in [28]. The first scheme uses a low quality reference (LQR) hash at the encoder. The LQR hash utilizes $W_{\mathrm{re}}$ frame to compute the residual (R) frame. The R frame is decomposed using discrete wavelet transform (DWT) followed by the Slepian-Wolf Set Partitioning in Hierarchical Trees [34] (SW-SPIHT) coding. The second scheme deploys the intra-decision mode technique along with SW-SPIHT coding. This scheme generates the $W_{\mathrm{re}}$ frame as weighted average interpolation of previous and next keyframes. The R frame is then computed and undergoes DWT decomposition, and the resulting coefficients are categorized into different modes. This scheme shows a small improvement in PSNR for Hall and Foreman video sequences compared to DISCOVER codec, albeit at the cost of high encoder computational complexity. The encoder exhibits the high complexity due to the computational cost of mode decision, DWT, SW-SPIHT and LQR. The DRVC for transform domain (TD) is presented in [35], where the R frame is computed by taking the difference between the current W frame and the previous keyframe. Subsequently, the scheme deployed a newly proposed quantization technique which is beneficial to achieve a low bit rate, especially for low motion videos. Most of the DC and AC coefficients in the residual frame have small intensity (value), especially for small motion. Therefore, conventional DVC quantization metrics do not assist in achieving any bit rate gain even after taking residual. Additionally, the correlation between the original and predicted residual transform coefficients is reduced. As a result, the channel decoder requires additional or extra parity bits to decode these transform coefficients, especially for the least significant bits. Thus, the achieved bit rate for decoding these residual transform coefficients becomes higher than that generated by coding the original frame. This indicates that the conventional DVC quantization is not suitable for the residual DCT coefficients because it does not assist in attaining compression efficiency even after taking the residual DCT coefficients.

## 3. Proposed DVRC Scheme

**Figure 1:**
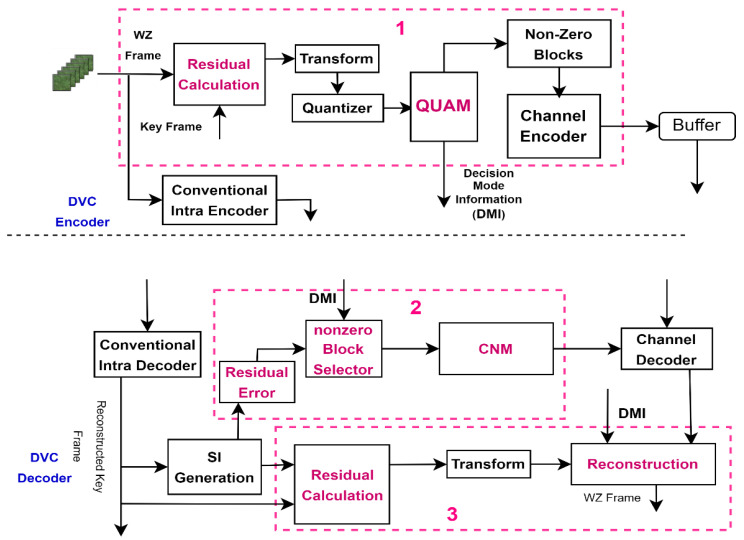
Proposed Quantized Transform Residual Decision Codec—QUATRID.

In this section, we present the proposed Quantized Transform Residual Decision (QUATRID) scheme. The QUATRID scheme, illustrated in Figure 1, introduces a set of novel features deployed within a baseline DRVC codec:

1. Residual Frame and QUAM Implementation
2. Correlation Noise Modelling for a Designed Scheme
3. Residual Frame Reconstruction

The new features of QUATRID introduced to the DRVC codec are marked in Figure 1 (entitled in dotted boxes 1, 2, and 3).

The QUATRID codec’s main encoder feature is QUantized TrAnsform Decision Mode (QUAM). The QUAM decides to skip or code any quantized transform 16 × 16 block (B_q16_). If B_q16_ is supposed to be coded, QUAM further processes it to extract the nonzero quantized transform 4 × 4 block (B_q4_). It generates the decision mode information (DMI) and passes the nonzero B_q4_ for channel encoding, while the DMI is sent to the decoder. The QUATRID decoder’s main feature is an online CNM, for which residual error is computed first. Then, based on DMI, CNM is calculated for corresponding nonzero blocks. The second important feature is the reconstruction of the R frame. First, based on DMI information, the full length decoded quantized transform bands are generated by combining the decoded quantized bin and blocks from SI correspond to skipped blocks for R frame reconstruction. Then, the reconstruction process for a transformed R frame is executed. The detailed implementation of each feature is discussed in the following subsections.

### 3.1. Residual Frame and QUAM Implementation

This subsection presents the details of R frame calculation and implementation of QUAM deployed at the encoder. The R frame calculation in the QUATRID scheme differs from most DRVC codecs. Instead of taking the difference between the current W frame and its estimated version $W_{\mathrm{re}}$, the R frame is computed by taking the difference between the current W frame and the previous frame. To derive the general mathematical notation, suppose that we have a video with N number of frames (I). Currently, we are at the k^th^ index frame (I_k_) then our previous frame is I_k−1_. Then mathematically, the R frame is presented in Equation (1), the x and y determine the position of any pixel in a frame.

(1) $$R\;\left(x,y\right)=I_{k}\left(x,y\right)-I_{k-1}\;\left(x,y\right)$$

Afterwards, the R frame is decomposed into the 16 × 16 blocks, which are then 4 × 4 block-wise transformed and quantized before the QUAM process. The QUATRID makes use of the quantization metric deployed in [35] for R frame quantization. However, the quantization step (W_q_) is computed differently and is presented in Equation (2). In Equation (2), the ${\left|C\right|}_{max}$ define the maximum absolute coefficient value of the corresponding transform band $b_{i}$ and $2^{M}$ defines the quantization level of the transformed band $b_{i}$. The i varies according to quantization metrics and goes from 1 to 16. This quantization step calculation leads to coarse quantization.

(2) $$W_{q}=\lceil \frac{2{\left|C\right|}_{max}}{2^{M-1}}\rceil$$

The QUAM is applied on each B_q16_, which determines whether it will be skipped or passed to the channel encoder. It generates the DMI and nonzero B_q4_, which are converted into bands. Finally, the bit planes are generated from these bands and channel encoded.

**Figure 2:**
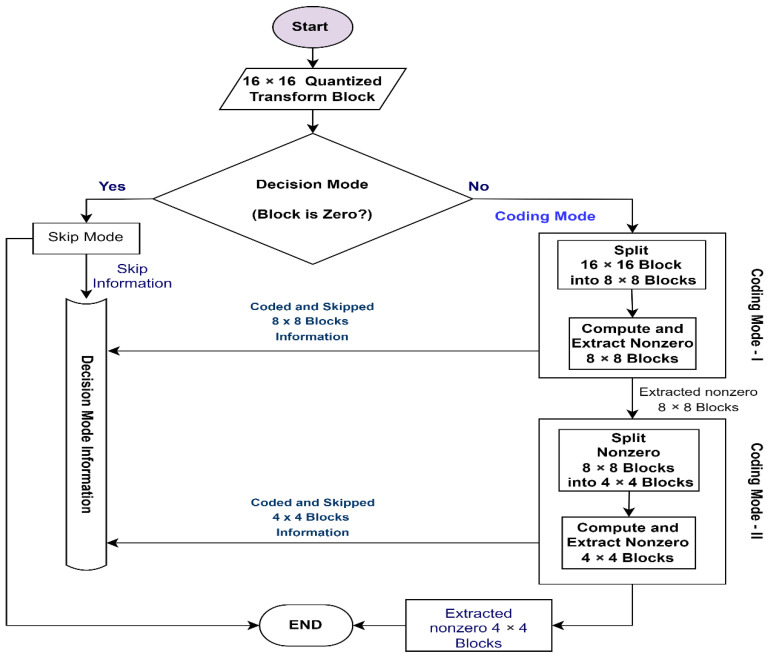
Flowchart for General Working of Proposed Quantized Transform Decision Mode (QUAM).

The flowchart in Figure 2 presents the general working scenario of QUAM. Starting with the B_q16_, at the first step, the Decision Mode (DM) checks whether the B_q16_ should be skipped or encoded. Based on the sum of absolute values within block B_q16_, as expressed in Equation (3), the decision information is passed to the DMI, where i and j determine the position of the quantized transform coefficient in B_q16_.

(3) $$\mathrm{Decision}\;\mathrm{Mode}=\{\begin{array}{c} \mathrm{Skip}\;\mathrm{Mode}\;\;\;\;\;\;\;\;\;\;\;\;\mathrm{if}\;\overset{16}{\underset{i,j=1}{\sum }}\left|B_{q16}\;\left(i,j\right)\right|=0\\ \mathrm{Coding}\;\mathrm{Mode}\;\;\;\;\;\;\;\;\;\mathrm{if}\;\overset{16}{\underset{i,j=1}{\sum }}\left|B_{q16}\;\left(i,j\right)\right|\neq 0 \end{array}$$

Equation (3) states that if the sum of the absolute of all values of the block B_q16_ is zero, then it will be skipped; otherwise, the coding mode will be activated. First, the coding mode-I (CM-I) will be activated in the coding mode process. The CM-I splits B_q16_ into 8 × 8 quantized transform blocks (B_q8_), where each B_q8_ block is tested using Equation (4).

(4) $$\mathrm{Coding}\;\mathrm{Mode}-I=\{\begin{array}{c} \mathrm{Coding}\;\;\;\;\;\;\;\;\;\;\mathrm{if}\;\overset{8}{\underset{p,z=1}{\sum }}\left|B_{q8}\left(p,z\right)\right|\neq 0\\ \mathrm{Skip}\;\;\;\;\;\;\;\;\;\;\;\;\;\mathrm{if}\;\overset{8}{\underset{p,z=1}{\sum }}\left|B_{q8}\left(p,z\right)\right|=0 \end{array}$$

Equation (4) defines that if the sum of the absolute of all coefficients of B_q8_ is not equal to zero, it is considered a nonzero block; otherwise, its skipped. The coding mode-I passes nonzero B_q8_ blocks to the coding mode-II (CM-II) for further processing. It also passes the code and skipped block information to DMI. In the CM-II process, the nonzero B_q8_ is first split into 4 × 4 quantized transform block (B_q4_). Then, it analyses each B_q4_ to sort out the nonzero B_q4_ and notifies the DMI about the coded and skipped blocks. Finally, the nonzero B_q4_ blocks are passed for the channel encoding process. The CM-II skips B_q4_ if the sum of the absolute of all coefficients of B_q4_ is equal to zero; otherwise, it is coded. Mathematically, the CM-II conditions are presented in Equation (5), where m and n determine the position of the quantized transform coefficient in B_q4_.

(5) $$\mathrm{Coding}\;\mathrm{Mode}-\mathrm{II}=\{\begin{array}{c} \mathrm{Coded}\;\;\;\;\;\;\;\;\;\;\;\;\;\;\;\;\;\;\;\;\;\mathrm{if}\;\overset{4}{\underset{m,n=1}{\sum }}\left|B_{q4}{}_{\;}\left(m,n\right)\right|\neq 0\\ \mathrm{Skipped}\;\;\;\;\;\;\;\;\;\;\;\;\;\;\;\;\mathrm{if}\;\overset{4}{\underset{m,n=1}{\sum }}\left|B_{q4}\left(m,n\right)\right|=0 \end{array}$$

### 3.2. Residual Error and Correlation Noise Model

The accurate online correlation noise model (CNM) improves error correction and coding efficiency. With an accurate online CNM, the channel decoder error correction capability improves, while demanding fewer parity bits from the channel encoder. In conventional DVC, the residual error, also known as noise residue, between the actual W frame and its estimated replica SI frame is required to calculate online CNM. In conventional DVC, the Laplacian distribution is normally used for modelling noise residue or residual error. In the DRVC codec, accurate residual error of the actual R frame and its replica Ŕ frame generated at the decoder is a quite challenging task. Therefore, it is hard to establish accurate online CNM to gain coding efficiency. In the proposed QUATRID scheme, the zero B_q16_ and B_q4_ blocks are skipped at the encoder and, therefore, it is required to establish the online CNM that fit the decoding of only the nonzero coded B_q4_. The following steps are taken to implement an online CNM that is the best fit for the QUATRID scheme. In the QUATRID decoder, the Ŕ (x,y) frame at the decoder is computed by subtracting the previously decoded frame from SI (x,y). To derive the mathematical notation, consider that our previous decoded frame is I_k−1_ (x,y), where the replica frame of the current I_k_ (x,y) frame generated at the decoder is SI (x,y). Then mathematically, Ŕ (x,y) is presented by Equation (6).

(6) $$Ŕ\;\left(x,y\right)=\mathrm{SI}\;\left(x,y\right)-{\;I}_{k-1}\left(x,y\right)$$

Since the original R (x,y) frame is not available at the decoder, the model is required to define the variance $\sigma^{2}$ between R (x,y) frame and corresponding estimated Ŕ (x,y) frame for estimation of Laplacian distribution parameter α. Therefore, we adopted the frame-level online α estimation, as described next.

First, the residual error frame Err_R_ (x,y) is computed. In an ideal case, when the original R (x,y) frame is available at the decoder, the Err_R_ (x,y) frame is computed by taking the difference between the original R (x,y) and Ŕ (x,y) frame. However, the R (x,y) frame is not available at the decoder; therefore, Err_R_ (x,y) frame is computed with the motion compensated version of $X_{B}$ and $X_{F}$ frames as follows, by Equation (7).

(7) $${\mathrm{Err}}_{R}\left(x,y\right)=\left|X_{B}\;\left(x+d_{x_{b}},y+d_{y_{b}}\right)-X_{F}\;\left(x+d_{x_{f}},y+d_{y_{f}}\right)\right|$$

$X_{F}\left(x+d_{x_{f}},y+d_{y_{f}}\right)$ and $X_{B}\left(x+d_{x_{b}},y+d_{y_{b}}\right)$ represent the forward and backward motion compensated frames, respectively. The (x,y) corresponds to a pixel location in the Err_R_ frame. The $\left(d_{x_{f}},d_{y_{f}}\right)$ and $\left(d_{x_{b}},d_{y_{b}}\right)$ represent the (horizontal and vertical) motion vectors for the $X_{F}$ and $X_{B}$ frames, respectively.

Based on DMI, the corresponding blocks are extracted from Err_R_ (x,y) as Ȓ (u,v). Then the variance ($\sigma_{Ȓ}^{2}$) is computed as follows

(8) $$\sigma_{Ȓ}^{2}=E\left[Ȓ{\left(u,v\right)}^{2}\right]-{\left(E\left[Ȓ\left(u,v\right)\right]\right)}^{2}$$

where u and v determine the position of a pixel in Ȓ. The variance $\sigma_{Ȓ}^{2}$ is a confidence measure of the Ŕ (x,y) frame creation process that indicates how good is the outcome of the frame interpolation process. Ideally, the $\sigma_{Ȓ}^{2}$ calculated in Equation (8) should be close to a variance of residual between actual R (x,y) and Ŕ (x,y). Since the actual R (x,y) is not available at the decoder, therefore, the variance ($\sigma_{Ȓ}^{2}$) computed in Equation (8) is a proposed way to represent the variance between the original R (x,y) and Ŕ (x,y), then, α is computed as presented in Equation (9)

(9) $$\alpha =\frac{\sqrt{2}}{\sigma_{Ȓ}^{2}}$$

Finally, the Laplacian distribution model for QUATRID is presented by Equation (10)

(10) $$p\;\left(Ȓ\left(u,v\right)\right)=\frac{\alpha }{2}e^{-\alpha \;|Ȓ\left(u,v\right)|}$$

### 3.3. Residual Frame Reconstruction

The decoded quantized bin is formed after channel decoding. Then, the DMI sent by the encoder is utilized to create the final full length quantized bin at the decoder for the reconstruction of the corresponding residual frame $\hat{R}$, which is further added up with the previous frame to get the final decoded Wyner-Ziv frame Ŵ.

The channel decoded quantized bin is converted into decoded B_q4_ blocks to generate the full length quantized bin. Based on the DMI, all decoded B_q16_ are generated by combining these decoded B_q4_ and zero 4 × 4 blocks (that represent skipped blocks). The decoded B_q16_ blocks, which include both skipped and non-skipped B_q16_, are converted into bands, and finally, the reconstruction of transform bands is performed. The reconstruction of each coefficient of the corresponding band is discussed in detail later in this section. After the successful reconstruction of all required bands, the 4 × 4 blocks are created. Finally, the decoded residual frame is formed after the inverse transformation of these blocks.

The accurate reconstruction of every single coefficient of the residual band plays a key role in the final decoded W frame. Generally, the decoded quantized bin consists of intervals (q) that are either zero (q = 0) or above zero (positive interval range, q > 0), or under zero (negative interval range, q < 0). The quantization process at the encoder introduces the quantization error. The reconstruction process assists in reconstructing values close enough to actual values by minimizing the quantization errors and leads to a better W frame reconstruction.

As mentioned earlier, the quantized bin consists of three types of intervals; zero, positive and negative. Generally, the transformed coefficient lies between the lower and upper bound ranges. For any positively transformed coefficient, the general lower bound is $q\times W_{q}$ and the upper bound is $\left(q+1\right)\times W_{q}$. For any negative transformed coefficient, the lower bound is $\left(q-1\right)\times W_{q}$ and the upper bound is $q\times W_{q}$ where q is negative. Some transformed coefficients also lie in $\left[-W_{q}{\;W}_{q}\right)$ so their upper bound is $W_{q}$ and lower bound is $-W_{q}$. During the quantization at the encoder, it is noticed that after the quantization, the positively transformed coefficients usually go toward the lower bound. Where after quantization, the negative values go toward the upper bound. Therefore, all three quantized interval conditions mentioned earlier are reconstructed with different algorithms to achieve significant reconstruction. Finally, the mathematical expressions are given in Equations (11)–(13).

If decoded quantized bin value (interval) q=0, it means that the actual transformed value was in the interval between $-W_{q}$ and $W_{q}$. Then reconstruction of a coefficient is performed by the different boundary conditions given in Equation (11), where $W_{q}$ defines quantization step, α Laplacian distribution parameter computed earlier.

(11) $$Û=\{\begin{array}{c} y+\sqrt{\frac{1}{\alpha }}-\frac{2\times W_{q}}{1-e^{2\times \alpha \times W_{q}}}\;\;\;\;,\;\;\;\;\;\;\;\;\;\;\;\;\;\;\;\;y<-W_{q}\;\;\\ \;y-\sqrt{\frac{1}{\alpha }}+\frac{2\times W_{q}}{1-e^{2\times \alpha \times W_{q}}}\;\;\;,\;\;\;\;\;\;\;y\in \left[-W_{q}{\;W}_{q}\right)\;\;\\ y-\sqrt{\frac{1}{\alpha }}-\frac{2\times W_{q}}{1-e^{2\times \alpha \times W_{q}}}\;\;\;,\;\;\;\;\;\;\;\;\;\;\;\;\;\;\;\;\;\;\;y\geq W_{q}\; \end{array}$$

In this case upper bound is $W_{q}$ and lower bound is $-W_{q}$. So, after quantization, for such a range, the resultant interval is q = 0; therefore, the boundary conditions defined by Equation (11) suit the reconstruction process. Generally, it is considered that when SI is of high quality, the α→∞, and vice versa. As α is changing from frame to frame, it contributes accordingly. When α is high, then it slightly moves the resultant reconstruction value and vice versa. Usually, when the actual transformed coefficient values are within $\left[-W_{q}\;0\right)$ and $\left[0\;\frac{W_{q}}{2}\right)$, the designed quantization slides them to zero. Therefore, second condition of Equation (11) shifts the y toward the lower bound range and reconstructs the improved quality coefficient Û. When y is out of the lower bound range, $y<-W_{q}$ the first condition of Equation (11) improves the reconstruction by bringing it within the range. Similarly, when y is equal or out of the upper bound range ($y\;\geq W_{q}$) then third condition of Equation (11) improves reconstruction by sliding it within the upper bound.

If q>0, then reconstruction is performed by different boundary conditions given in Equation (12). If decoded quantized interval value is greater than zero (q>0), it determines that the actual transformed coefficient encoded at the encoder lies in positive interval ranges.

(12) $$Û=\{\begin{array}{c} y+\sqrt{\frac{1}{\alpha }}-\frac{2\times W_{q}}{1-e^{2\times \alpha \times W_{q}}},\;\;\;\;\;\;\;\;\;\;\begin{array}{c} \;y\in \left[q\times W_{q}\;\left(q+1\right)\times W_{q}\right)\;\\ \mathrm{and}\;y<q\times W_{q} \end{array}\;\\ y+\frac{1}{\alpha }-\frac{2\times W_{q}}{1-e^{2\times \alpha \times W_{q}}}\;,\;\;\;\;\;\;\;\;\;\;\;\;y\geq \left(q+1\right)\times W_{q}\; \end{array}$$

Equation (12) is used to reconstruct the coefficient that belongs to the positive intervals with lower bound $q\times W_{q}$ and upper bound $(q+1)\times W_{q}$. When SI’s coefficient y lies within $\left[q\times W_{q},\;\left(q+1\right)\times W_{q}\right)$ or it is under the lower bound, then first condition of Equation (12) enhances the reconstruction quality by sliding it to the upper bound side. To improve the reconstruction quality when SI’s coefficient y lies out of the upper bound $\left(q+1\right)\times W_{q}$ then second condition of Equation (12) reconstruct the enhanced quality coefficient by bringing it within the upper bound.

If q<0, then reconstruction is performed by different boundary conditions given in Equation (13). If decoded quantized interval value is less than zero (q<0), it determines that the actual encoded transform coefficient lies in negative interval ranges.

(13) $$Û=\{\begin{array}{c} y+\frac{1}{\alpha }-\frac{2\times W_{q}}{1-e^{2\times \alpha \times W_{q}}},\;\;\;\;\;\;\;\;\;\;\;\;\;\;\;\;\;\;\;\;\;\;\;\;y<\left(q-1\right)\times W_{q}\;\\ y-\frac{1}{\alpha }-\frac{2\times W_{q}}{1-e^{2\times \alpha \times W_{q}}},\;\;\;\;\;\;\;\;\;\;y\in \left[\left(q-1\right)\times W_{q}\;q\times W_{q}\right)\;\\ y-\frac{1}{\alpha }+\frac{2\times W_{q}}{1-e^{2\times \alpha \times W_{q}}},\;\;\;\;\;\;\;\;\;\;\;\;\;\;\;\;\;\;\;\;\;\;\;\;\;\;\;\;\;\;\;\;\;\;\;\;\;y\geq q\times W_{q}\;\; \end{array}$$

Equation (13) is used to reconstruct the coefficient that belongs to the positive intervals with lower bound $\left(q-1\right)\times W_{q}$ and upper bound $q\times W_{q}$. Usually, when actual transformed coefficient values are within $\left[\left(q-1\right)\times W_{q}\;q\times W_{q}\right)$ especially close to the lower bound, the designed quantization function slides intervals to the upper bound side. Therefore, deploying a second condition of Equation (13) shifts the y toward the lower bound range and reconstructs the improved quality coefficient Û. When y is out of the lower bound range, $\left(q-1\right)\times W_{q}$ the first condition of Equation (13) improves the reconstruction by bringing it within the range. Similarly, when y is equal or out of the upper bound range ($q\times W_{q}$) then third condition of Equation (13) improves reconstruction by sliding it below the upper bound.

To briefly conclude the proposed QUATRID, its primary attributes are:

- QUAM for DRVC that drops the zero QT blocks of the residual frame. QUAM generates fewer bit planes to be channel encoded, thus reducing the complexity of the channel encoding. Similarly, the fewer channel-encoded bit planes reduce the complexity of channel decoding. Therefore, QUAM reduces the overall computational complexity while improving coding efficiency.
- Online correlation noise model (CNM) at the decoder to perform the error correction of the bit planes generated using a limited number of nonzero QT blocks. The decision mode information (DMI) is utilised for CNM.
- A reconstruction algorithm for the final reconstructed WZ frame reconstruction. The algorithm combines the blocks taken from SI based on decision mode information provided by the encoder and decoded quantized blocks. The primary contribution is designing an algorithm for reconstructing residual frames from a set of combined SI blocks and decoded quantized blocks.

## 4. Experimental Results and Analysis

The experiments are carried out in MATLAB 2018b on an Intel Core-i7-7820HQ CPU 2.90 and operating system (OS) Windows 10 (64-bits) system. The performance of the codec is presented in the average computational time of partial WZ encoding time (T_p_), the average computational time of full WZ encoding (T_f_), the average computational time of channel encoding (T_c_), the average number of encoded bit planes (NBP) per frame and rate-distortion (RD) performance of video sequence. The test video sequences utilized for performance are Hall, Foreman, and Coastguard with a frame rate (fps) of 15 Hz and video size of 176 × 144. For DIS and QUATRID codecs, the group of pictures (GOP) size 2 is adopted, meaning one frame is called a keyframe (KF), and the other is a Wyner-Ziv frame. The quantization parameter (Qp) is deployed for KF quantization. The quantization metric (Qm) is used for the quantization of the W frame in the DIS codec and the residual frame in QUATRID codec. The test conditions (RD points) provided by DIS are utilized for Hall, Foreman, and Coastguard. The RD performance of QUATRID is compared with conventional DVC (DIS) codec and conventional Intra H.264/AVC codec.

**Figure 3:**
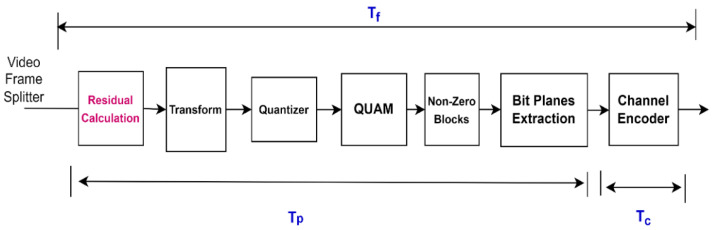
Computational Times of WZ Encoder and Channel Encoder of QUATRID codec.

Figure 3 shows the computational times of different components of the WZ Encoder, such as the average partial computational time of WZ encoding (T_p_), the average full computational time of WZ encoding (T_f_), and the average computational time of channel encoding (T_c_). These computational times are measured for different videos with different motions.

Table 1 provides a detailed comparison of T_p_ and T_f_ of the DIS and QUATRID codec for Hall, Foreman, and Coastguard video sequences with GOP size 2. The components whose T_p_ and T_f_ are measured for the QUATRID codec are shown in Figure 3. In Table 1, the Partial Computational Time Improvement Ratio (CTIR_p_) and full Computational Time Improvement Ratio (CTIR_f_) define the improvement ratio in average computational time by the QUATRID codec compared to DIS and are calculated by Equations (14) and (15). The T_p,DIS_ and T_p,QUATRID_ defines the T_p_ of DIS and QUATRID, respectively. The T_f,DIS_ and T_f,QUATRID_ defines the T_f_ of DIS and QUATRID.

(14) $${\mathrm{CTIR}}_{p}=\frac{T_{p,\mathrm{DIS}}}{T_{p,\mathrm{QUATRID}}}$$

(15) $${\mathrm{CTIR}}_{f}=\frac{T_{f,\mathrm{DIS}}}{T_{f,\mathrm{QUATRID}}}$$

The CTIR_p_ of Hall for low RD points is less than DIS because the T_p_ of the QUATRID is a bit higher than DIS. The T_p_ of the QUATRID is a bit high due to the computation of the QUAM process. The overall T_f_ of QUATRID is less than the T_f_ of the DIS because fewer NBP is channel encoded. Therefore, CTIR_f_ is high for these low RD points. Further, the CTIR_p_ reached up to 1.74 folds for high RD points. In addition to this, for such high RD points, the CTIR_f_ is also high, which determines that QUATRID has low computational complexity. The CTIR_f_ varies from 4.5 to 9.32 folds which determines that the computational complexity of QUATRID encoders is 4.5 to 9.3 times less than the DIS. The CTIR_p_ for high RD points ranges from 1.25 to 1.74 folds. Therefore, the QUATRID performs 1.25 to 1.74 folds faster than DIS. At these points, the number of bands to be quantized increases and is further processed; therefore, DIS exhibits high computational complexity because it needs to perform band organization and bit-plane extraction for all the blocks. Where in the QUATRID codec, most of the zero B_q16_ and B_q4_ are dropped during the QUAM process; therefore, fewer bit planes are generated with the remaining nonzero B_q4_. These fewer bit planes are encoded quickly; therefore, due to less channel coding computational time, the CTIR_f_ is increased. Further, for the RD points where the T_p_ of QUATRID was high due to the QUAM process, the T_f_ of QUATRID remains less than DIS because few bit planes are channel encoded. Therefore, skipping the zero B_q16_ and B_q4_ blocks assists in reducing the channel encoding process. It also reduced the channel decoding process because fewer bit planes are decoded. Thus, low computational channel coding (encoding and decoding) is achieved with the QUAM process deployed on the DRVC codec.

The T_p_, T_f_, CTIR_p_, and CTIR_f_ values for both Foreman and Coastguard are also shown in Table 1. Due to the QUAM process, T_p,QUATRID_ is higher than T_p,DIS_ for low RD points, resulting in a decrease in CTIR_p_. However, at high RD points, the T_p,QUATRID_ is smaller than T_p,DIS_ because DIS requires more time to generate bit planes, resulting in an increase in CTIR_p_ for both sequences. The table demonstrates that Foreman’s CTIR_p_ ranges from 0.53 to 1.43 folds, whereas, for Coastguard, it ranges between 0.6 and 1.87 folds. Further study of the findings indicates that for all RD points, T_f, QUATRID_ is smaller than T_f, DIS_ because the QUATRID channel encodes fewer bit planes, increasing CTIR_f_. The high CTIR_f_ determines that the computational complexity is reduced by the pre-mentioned times. Based on the table, Foreman and Coastguard’s CTIR_f_ ranges from 3.75 to 5.96 folds and 5.38 to 7.59 folds, respectively.

The average computational time of channel encoding (T_C_) and the average number of bit planes (NBP) are provided in Table 2. The CTIR_C_ defines channel encoding time efficiency. It is defined as the percentage improvement in T_C_ taken by the QUATRID codec to T_C_ taken by DIS. The BPR defines the percentage bit plane reduction. The CTIR_C_ and BPR are calculated by Equations (16) and (17), respectively. In Equation (16), the T_C,DIS_ and T_p,QUATRID_ defines the T_C_ of DIS and QUATRID, respectively. In Equation (17), NBP_DIS_ and NBP_QUATRID_ define the NBP of DIS and NBP of QUATRID codecs, respectively.

(16) $${\mathrm{CTIR}}_{C}=\frac{T_{C,\mathrm{DIS}}}{T_{C,\mathrm{QUATRID}}}$$

(17) $$\mathrm{BPR}=\frac{{\mathrm{NBP}}_{\mathrm{DIS}}-{\;\mathrm{NBP}}_{\mathrm{QUATRID}}}{{\mathrm{NBP}}_{\mathrm{DIS}}}\times 100$$

Table 2 results depict the average computational time of channel encoding (T_C_) and an average number of bit planes for Hall, Foreman, and Coastguard video sequences. The results illustrate that for all the RD points, the channel encoding time taken by the DIS is comparatively higher than the time taken by the QUATRID codec for all video sequences. The T_C,DIS_ is high because many bit planes are required to be channel encoded. However, the QUATRID codec has fewer bit planes to be channel encoded, achieving the high CTIR_C_ for all RD points. The table analysis for each video illustrates that Hall’s CTIR_C_ ranges from 7 to 33 folds. The high CTIR_C_ indicates that the computational cost of channel coding is reduced by the stated factor. This CTIR_C_ is too high for low RD points because fewer bit planes are channel encoded. For high RD points, the CTIR_C_ is slightly reduced because NBP increased. The bit plane reduction percentage is computed to determine the performance of QUATRID in terms of the capability to reduce the bit planes. The high BPR determines that a large number of bit planes are reduced, and a small BPR determines a smaller number of bit planes is reduced. The high BPR also determines that the T_C_ is smaller and vice versa. This high BPR also shows that channel decoding computation complexity is smaller because fewer NBP is decoded. The QUATRID encoded significantly less NBP compared to DIS. Thus, the BPR ranges from 84% to 97% for Hall, which is a major advantage of incorporating QUAM.

In addition, the high BPR determines the low channel coding computational complexity. Further analysis of the table for Foreman leads to the conclusion that the T_C,QUATRID_ is comparatively smaller than T_C,DIS_ because fewer NBP are channel encoded. Consequently, QUATRID acquired a high CTIR_C_, which varies from 4.8 to 22.69 folds and BPR ranges from 78% to 95.6%. Similar to the other sequences, the QUATRID shows the same T_C_ and NBP findings for Coastguard. Thus, Coastguard CTIR_C_ ranges between 6.7 and 34 folds, while BPR ranges between 75% and 96.95%.

Table 1 and Table 2 analysis conclude that instead of adding any computational complexity, the QUAM process improves the CTIRp, CTIRC, and CTIR_f_ of the QUATRID. In addition, QUAM assists in reducing the channel decoding process.

Table 3 shows the RD performance of DIS, Intra, and QUATRID codecs for the Foreman video sequence with a GOP size of two. The results show that the QUATRID has shown dominance in coding efficiency. The evaluation of experimental results indicates that the coding efficiency of QUATRID over DIS ranges from 13.6 kbps to 42 kbps. Further analysis shows that for low RD points, At low RD points, the coding efficiency attained by the QUATRID compared to DIS ranged from 19.32 kbps to 26 kbps while QUATRID encoder computation was reduced by more than four times. While it costs only in the degradation of the PSNR up to 0.26. Further, at high RD points, the coding efficiency achieved by QUATRID was up to 42.11 kbps with slight PSNR degradation of up to 1.55 dB. However, the QUATRID computational complexity analysis shows low computational complexity for these RD points. Further NBP analysis shows that fewer channel encoded bit planes are utilized for all RD points and still achieve the comparable PSNR. The analysis evidenced that the BPR is up to 78% for the highest RD point. In addition to this, for the same RD point, the CTIR_f_ is more than 3.75 times and goes up to more than 5 times. Further, the BPR ranges from 78% to more than 95%. In addition to this, coding efficiency achieved by QUATRID in comparison to Intra varied from 27.74 kbps to 30.22 kbps for low RD points, while PSNR improvement of 0.81 dB was observed. However, at high RD points, it ranged from 9 kbps to 30.66 kbps with a PSNR degradation of 1.33 dB. Based on the results analysis and discussion, it can be generalized that QUATRID obtained high coding efficiency for all RD points than DIS and Intra. In contrast to DIS, QUATRID decreased the computational complexity of the encoder and the computational complexity of channel coding, while resulting in a modest PSNR degradation. The results evidenced that the later bands have few nonzero values due to coarse quantization in high RD points. Even the intensity of those values is too small, so they do not contribute much to reconstruction. Therefore, the reconstructed frame quality could not improve on a large scale due to these mostly zero values. However, for the frames which have a considerable count of nonzero B_q4_, the reconstruction process improves their quality on a large scale.

Table 4 illustrates the RD performance results comparison of DIS, Intra, and QUATRID codecs for Coastguard video sequence with GOP 2. The results indicate that QUATRID coding efficiency varies from 12.01 kbps to 38.05 kbps. Further, the results analysis indicates that QUATRID coding efficiency varies from 16.56 kbps to 22.08 kbps for the low RD points. In addition, the coding efficiency varies between 12.01 kbps and 38.05 kbps at high RD points. Further, in comparison to Intra codec, the QUATRID coding efficiency remains better for all RD points. The results analysis demonstrates that the QUATRID is coding efficient at low RD points by saving the coding rate ranges between 49.8 kbps and 55.34 kbps. Whereas, at high RD points QUATRID saved coding rate ranges from 6.45 kbps to 68.86 kbps. Further analysis directs that for low RD points, the RD performance is comparable with DIS since PSNR is relatively close to the DIS. Especially for the lowest RD point, the QUATRID increased the PSNR gain up to 0.07 dB over DIS. However, for intermediate and high RD points, it lags from 0.05 to 0.93 dB. In contrast, the QUATRID is much better and outperformed the Intra by gaining the PSNR ranging between 0.51 dB and 0.58 dB for low RD points. Furthermore, the QUATRID performance is quite close to Intra at intermediate RD points with a PSNR gain of 0.04 dB; however, it lagged at high RD points as PSNR degradation ranges up to 0.26 dB. While coding efficiency is far better than both Intra and DIS for all RD points. Close inspection of the results at the bands level reveals that most of the values in later bands have very few nonzero values due to coarse quantization. Even these values have small intensity. Therefore, reconstructing these dead zone values of later bands is challenging. Therefore, these zero values could not improve the reconstructed frame quality on a large scale. However, for the frames with a considerable count of nonzero B_q4_, the reconstruction process improves their quality on a large scale. Although the QUATRID PSNR slightly degraded at high RD points, the coding efficiency is too high throughout all RD points. Other major performance advantages of QUTRID are the CTIR_C_, CTIR_f_, and BPR. The achieved CTIR_C_ varies from 6.7 to 34 folds. In addition to this, the CTIR_f_ varies from 5.2 to 7.6 folds. In addition to this, the BPR achieved by QUATRID is 85% to 97%.

Table 5 determines the rate-distortion (RD) performance of DIS, Intra (Conventional Codec), and QUATRID codec. The RD performance table for the low motion Hall video sequence determines that the QUATRID codec achieved high coding efficiency for low RD points, whereas its PSNR slightly decreased. This video sequence is of low motion and coded at a low bit rate even at high RD points. Therefore, DMI generated by the QUATRID becomes an additional burden at high RD points and increases the coding rate. The coding efficiency gained by the QUATRID codec varies from 6.56 kbps to 10.65 kbps. However, the QUATRID codec lagged in coding efficiency for high RD points compared to DIS. At the same time, the QUATRID codec is much more coding efficient than Intra. For the RD points where the QUATRID codec is not enough coding efficiently as DIS, the number of nonzero B_q4_ per frame was increased. Once the number of nonzero B_q4_ increased in a frame, the DMI bits rapidly increased, and it affected the overall coding efficiency for corresponding RD points. Furthermore, after the QUAM process, most of the frames generated a small number of nonzero B_q4_. Thus, fewer bit planes are generated and encoded. The reconstruction of such frames improves the reconstructed frame quality, but its effect is smaller than in other reconstructed frames with more nonzero B_q4_. The band level evaluation of such frames determines that later bands of the frame have few nonzero values due to coarse quantization, and the intensity of these nonzero values is small. Therefore, the reconstructed frame quality could not improve on a large scale due to these mostly zero values. For the frames which have a considerable count of nonzero B_q4_, the reconstruction process improves their quality on a large scale. The PSNR analysis shows that the QUATRID outperforms the DIS by 0.06 dB for low RD points. For intermediate RD points, it is quite comparable with DIS. However, for high RD points, it lagged up to 0.82 dB. It is evident that for low RD points, the performance of QUATRID is 0.10 dB better than Intra at the lowest RD point. While for other low RD points, it is comparable with Intra codec. However, the QUATRID lagged up to 1.11 dB for high RD points. The major advantage of QUATRID is CTIR_C_, CTIR_f_, and BPR for these high RD points. The CTIR_C_ and CTIR_f_ are 7.9 and 5.85 folds, respectively. The BPR by the QUATRID codec is up to 84% for these high RD points. The maximum BPR achieved by QUATRID for Hall is 97% at low RD points.

**Figure 4:**
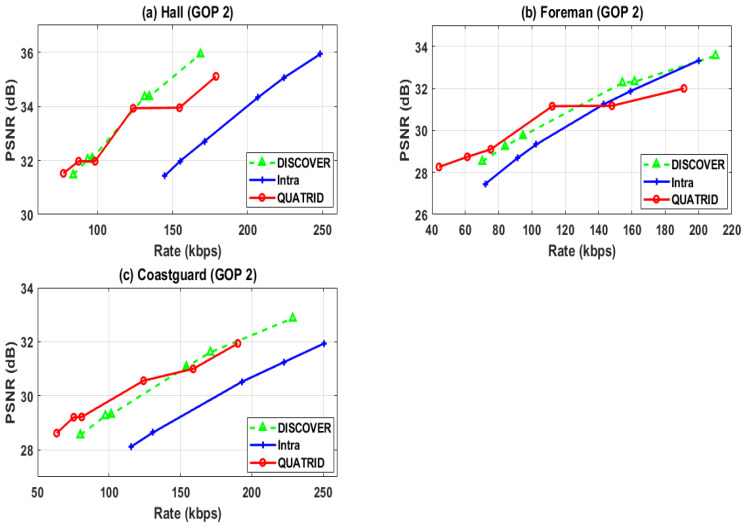
Rate-Distortion (RD) Performance Evaluation of Different Video Sequences.

Figure 4 shows the RD performance of different video sequences with GOP 2 for DIS, Intra, and QUATRID. Figure 4a shows the RD performance of the Hall video sequence. The graph analysis shows that the QUATRID performance is comparable with DIS for low RD points; however, it outperformed the Intra codec. Further investigation determines that QUATRID performance slightly lagged compared to the DIS for intermediate RD points, but slightly improved over the Intra codec. The RD performance QUATRID at high RD points lagged compared to both DIS and Intra codec. However, close analysis determines that the major advantages of QUATRID achieved throughout all RD points are high coding efficiency, low encoder computational complexity, and less channel coding process. The coding efficiency varies from 6.02 kbps to 7.65 kbps. In addition to this, the QUATRID computational complexity analysis determines low computational complexity for all RD points. Further NBP analysis shows that fewer channel encoded bit planes are utilized for all RD points and can still achieve the comparable PSNR. The analysis is evident that the BPR is up to 84% for the highest RD point. In addition to this, for the same RD point, the CTIR_f_ is more than 5.8 folds, which determines that QUATRID performed 5.8 times faster than QUATRID. Although QUATRID slightly lagged in PSNR gain at some RD points, channel encoding computational complexity was reduced by more than fivefold because the BPR ranged to 84%. Further, QUATRID gains coding efficiency compared to Intra, which varies from 64 to 83 kbps. Despite its modest coding efficiency for Hall video, the main benefit of QUATRID is its lower computational cost for channel encoding compared to the DIS codec. Figure 4b shows the RD performance of the Foreman video sequence. The QUATRID codec at low rate points shows better RD performance than DIS and Intra. It achieved the gain in PSNR. While for intermediate rate points, the QUATRID codec lagged from the DIS. However, it outperforms the Intra. The QUATRID codec PSNR performance marginally degraded for the high rate point from both the DIS and Intra. However, the BPR analysis shows that the QUATRID BPR is up to 78% for such a high rate point. In addition to this, for the same RD point, the CTIR_f_ is more than 3.75 times and goes up to more than five times. In Figure 4, graph (c) shows the RD performance of the Coastguard video sequence. The QUATRID outperforms low rate points and gains up to 0.07 dB PSNR. The QUATRID’s RD performance is comparable with the DIS code for intermediate rate points. In addition to this, it is better from the Intra codec. Comparing the performance at intermediate rate points, QUATRID is comparable to DIS and superior to Intra. However, the QUATRID RD performance slightly lagged from the DIS at high rate points. However, the QUATRID depicts low computational complexity and comparable PSNR gain for Coastguard, as it does for other sequences. The analysis evidenced that the BPR is up to 85% for the highest RD point. Moreover, for the same RD point, the CTIR_f_ is more than five folds, indicating that QUATRID performs 5.38 to 7.59 times faster than the DIS codec.

The performance graphs show that the QUATRID performance in terms of rate for all the video sequences is very good. The coding rate for all the RD points for all video sequences is less than DIS and Intra, so this depicts the high coding efficiency offered by the QUATRID for all RD points for all video sequences. The coding efficiency achieved by QUATRID for the Hall video sequence varies from 6.02 kbps to 7.65 kbps. The Hall video is a low motion video, and from the results of DIS, we can see that its coding rate is lower than other video sequences. Therefore, QUATRID achieved up to 10.65 kbps coding efficiency. The RD performance graph indicates that QUATRID is coding efficient throughout the RD points of the Foreman video sequence. The coding efficiency improvement over DIS, achieved by QUATRID, varies from 13.6 kbps to 42.11 kbps. The coding rate of QUATRID for Coastguard is lower than DIS for all RD points, which means coding efficiency is improved. The coding efficiency achieved by QUATRID varies from 12.01 kbps to 38.05 kbps.

Table 6 determines the average feedback channel requests per frame when channel coding (LDPCA) is deployed with DIS and with QUATRID. These average feedback request results are for the highest RD points for all video sequences. The evaluating the performance by average feedback requests per frame, the performance of the QUATRID is far better than the DIS for all video sequences. The average feedback requests reduction per frame occurs because QUATRID has less number of bit planes per frame compared to a DIS codec. However, the result evaluation determines that the average feedback requests per bit plane of the DIS for the Hall and Foreman sequence are slightly less than the QUATRID. However, due to the proposed CNM efficiency, the average feedback per bit plane of the QUATRID is far better than the DIS for the Coastguard sequence. The feedback channel requests reduction ratio is obtained by dividing the feedback channel requests of DIS by QUATRID’s feedback channel requests to compare QUATRID and DIS performance. The high feedback channel requests reduction ratio (higher than 1) demonstrates that QUATRID efficiently minimises the feedback requests compared to DIS. Furthermore, the high feedback request reduction ratio shows that latency drops proportionally to that degree. The QUATRID feedback requests reduction ratios for Hall, Foreman, and Coastguard are 2.8, 3.2, and 4 folds, respectively, demonstrating that QUTRID feedback requests are reduced to that extent. The latency is also reduced accordingly. The average feedback requests are reduced because of the CNM model and also because fewer bit planes are coded.

Table 7 summarizes the performance comparison of DIS, QUATRID, and Intra codecs. A comparison of the computational complexity of QUATRID with DIS is carried out by estimating computational time improvement ratios (CTIR_p_, CTIR_f_, CTIR_C_) by Equations (14)–(16). Furthermore, codec performance is evaluated in terms of the average number of bit planes (NBP) per frame, and performance comparison of the QUATRID and DIS is conducted by bit plane reduction percentage (BPR). In addition to this, Bjøntegaard delta performance is computed to evaluate the coding efficiency and quality achievement of QUATRID compared to DIS and Intra codecs.

Further, in Table 7, the computational complexity performance comparison of QUATRID and DIS is evaluated in terms of average full encoding computational time improvement ratio (CTIR_f_) and average channel coding computational time improvement ratio (CTIR_C_) for Hall, Foreman, and Coastguard. The high CTIR_f_ and CTIR_C_ establish that QUATRID has a fast computation, indicating the low computational complexity of QUATRID compared to DIS. The CTIR_f_ of Hall, Foreman, and Coastguard varied from 4.5 to 9.3 folds, 3.7 to 5.9 folds, and 5.2 to 7.5 folds, respectively. This establishes that the QUATRID encoder performed the pre-mentioned times faster than DIS for mentioned videos. While CTIR_C_ of Hall, Foreman, and Coastguard varied from 7 to 33 folds, 4.8 to 22.6, and 8.7 to 34 folds. This establishes that the QUATRID channel encoder performed the pre-mentioned times faster than DIS’s channel encoder for mentioned videos. The QUATRID channel encoding process is fast because it has to encode fewer bit planes. The QUAM deployed with the QUATRID encoder dropped a large number of zero quantized transform blocks of sizes 16 × 16 and 4 × 4, leading to fewer bit planes with remaining nonzero blocks. The bit plane reduction percentage (BPR) computed between an average number of bit planes (NBP) encoded by DIS and QUATRID determines the percentage reduction in channel encoded bit planes of QUATRID. The high BPR percentage determines that relatively few bit planes are channel encoded using QUATRID, which leads to high CTIR_C_. The BPR of Hall, Foreman, and Coastguard varied from 84% to 97%, 78% to 95%, and 86% to 96%, respectively. The CTIR_f_, CTIR_C_, and BPR analysis indicate that QUATRID full encoding, channel encoding computational complexity is reduced to a great extent and where high BPR percentage indicates that the overall channel coding (encoding and decoding) process is reduced with the deployment of QUAM.

Further, Table 7 determines the Bjøntegaard delta performance, deployed to measure the QUATRID’s performance for different video sequences. The RD performance defines the codec’s performance in terms of coding rate and PSNR achievement at different RD points. The evaluation of the RD findings of Hall, Foreman, and Coastguard demonstrates that QUATRID has achieved a high level of coding efficiency throughout all RD points, where PSNR improved at certain RD points and slightly reduced at other RD points. The coding efficiency identified remains 6 to 7 kbps, 13 to 42 kbps, and 12 to 38 kbps for Hall, Foreman, and Coastguard, respectively. The RD performance is used as a quality evaluation tool and shows the PSNR dependency on the bit rate scale. It evaluates which codec performs better in PSNR (or reduced distortion effectively) at different bit rates. From RD curves, we can differentiate which codec performs superior at the given rate points. However, the Bjøntegaard delta (BD) model is used to compute the average PSNR and bit rate differences between two RD curves of the different codecs. Bjøntegaard delta analysis differentiates these RD curves by computing a single number or point between two RD plots, which tells almost everything. The Bjøntegaard delta metric computation contains two parts—BD PSNR (also written as B-DSNR) and BD Rate (B-DBR). Computing the Bjøntegaard Delta metric and its evolution offer a good comparison of the RD performance of two different codecs. Both BD Rate and BD PSNR are interpreted individually and differently. The BD Rate indicates the number of bits saved (coding efficiency) by the test codec in comparison to the reference codec while keeping the same PSNR.

The detailed profiling of the BD Rate calculated with DIS and QUATRID RD curves of Hall, Foreman, and Coastguard identified that QUATRID is coding efficient and capable of saving the bit rate between 5.4% and 10.48% while gaining the same PSNR as the DIS, except for Hall video sequences. For this low motion video, QUATRID demands a 4.2% bit rate to achieve the same PSNR. Therefore, break down these RD curves into low and high RD points and analyze each separately. This breakdown analysis for BD Rate shows that QUATRID effectively saves a high bit rate at low RD points for all video sequences compared to high RD points where it slightly lags in PSNR gain. The QUATRID shows coding efficiency by saving 2.7%, 15.5%, and 18.4% for Hall, Foreman, and Coastguard, respectively, while maintaining the same PSNR. The BD Rate calculated with Intra and QUATRID RD curves of Hall, Foreman, and Coastguard identified that QUATRID is coding efficient by saving the bit rate between 18.86% and 37.41% while gaining the same PSNR as the Intra. Finally, thorough profiling of BD PSNR calculated with DIS and QUATRID RD curves of Hall, Foreman, and Coastguard identified that QUATRID can gain the PSNR. The QUATRID PSNR gain varies from 0.061 dB to 0.32 dB while utilizing the same bit rate, except for the Hall video sequence, for which it lost 0.23 dB PSNR. Similarly, such as BD Rate analysis, break down these RD curves into low and high RD points and analyze each separately. This breakdown analysis for BD PSNR identifies that at a low RD point, QUATRID shows the capability of gaining a PSNR for all video sequences. The BD PSNR for Hall, Foreman, and Coastguard directed that QUATRID gain the PSNR 0.08 dB, 0.44 dB, and 0.68 dB, respectively, over DIS. Furthermore, the BD PSNR calculated with Intra and QUATRID RD curves of Hall, Foreman, and Coastguard identified that QUATRID gains 2.31 dB, 0.69 dB, and 1.74 dB PSNR, respectively.

Table 8 illustrate the RD performance comparison of Intra and QUATIRD Codecs for additional new test sequences; Akiyo and Salesman. The performance of QUATRID is compared only with the Intra codec. The test video sequences exhibit medium to high motion in some parts of the frame, which means motion changes from frame to frame in regular intervals of time with medium or high speed. Table analysis shows that for the Akiyo test sequence, the QUATRID codec achieves the coding efficiency from 80.15 kbps to 116.33 kbps over the Intra codec. Further, the PSNR gain of the QUATRID codec is from 0.26 dB to 1.33 dB. Further, the Salesman results evaluation unfolds that the QUATRID achieves coding efficiency from 123.33 kbps to 192.14 kbps over the Intra codec. In this video sequence, the QUATRID gains 0.13 dB to 0.73 dB for low RD points. The PSNR performance for high RD points was slightly degraded due to the coarse quantization of the last few AC bands. The coarse quantization generates a small number of low-intensity nonzero values.

Table 9 results depict the average computational time of full Wyner-Ziv encoding, channel encoding (T_C_), and an average number of bit planes (NBP) for BUS and Coastguard video sequences with 352 × 288 resolution. The evaluation of results taken under random conditions determines that the average computational complexity of full Wyner-Ziv encoding (T_f_) and channel encoding (T_C_) of QUATRID is far better than DIS. The CTIR_f_ and CTIR_C_ of both sequences determine that QUATRID performs 1.17 to 1.7 times faster than DIS. Further analysis of a number of bit plane results depicts that QUATRID efficiently reduces 16.05 to 57.5 percent of channel encoding bit planes. Therefore, be able to reduce the channel encoding computational complexity, which directly reduces the channel decoding process.

## 5. Conclusions

The most often cited benefit of DVC is the low complexity encoder. To decrease the burden of the channel coding process, achieve coding efficiency, and keep encoder complexity low, this research article proposed DRVC based scheme named QUATRID. The QUATRID codec first deploys a decision mode called the QUAM at the encoder, which drops the zero quantized transform blocks. Therefore, fewer bit planes are generated that are required to be channel encoded. This reduced both the channel encoding and decoding processes. Secondly, an online CNM is deployed at the decoder to attain maximum coding efficiency during the channel decoding process for the proposed scheme. Finally, a new reconstruction method is adopted to reconstruct the encoded residual frame and the final WZ reconstructed frame. Doing so resulted in the QUATRID achieving better RD performance than DIS and Intra codecs. The thorough profiling of the QUATRID for coding efficiency and quality analysis and comparison concludes that QUATRID significantly achieves high coding efficiency and PSNR while substantially reducing the computational complexity. Bjøntegaard delta analysis shows that QUATRID successfully achieved 5.4% to 10.48% coding efficiency and 0.06 dB to 0.32 dB PSNR gain while reducing the encoder, channel coding computational complexity, BPR, and feedback requests reduction ratio by 3.7 to 9.3 folds, 4.8 to 34 folds, 84% to 97%, and 2.8 to 4 folds, respectively, in comparison to DISCOVER. Moreover, in comparison to Intra codec, QUATRID successfully saved coding rates from 18.86% to 37.41% and gained PSNR up to 0.08 dB to 0.68 dB.

## Figures and Tables

**Table 1:** Average Computational Time of Partial WZ Encoding (T_p_) and Full WZ Encoding (T_f_) per frame of DIS and QUATRID Codec for Hall, Foreman, and Coastguard Video Sequence for GOP 2.

Video Details			T_p_ (sec)			T_f_ (sec)		
Name	Qp	Qm	DIS	QUATRID	CTIR_p_	DIS	QUATRID	CTIR_f_
Hall	37	1	0.041	0.0849	0.48	0.437	0.0967	4.52
	36	2	0.044	0.0847	0.52	0.495	0.1053	4.70
	36	3	0.067	0.0875	0.77	0.751	0.1305	5.76
	33	4	0.110	0.0878	1.25	1.362	0.1545	9.33
	33	5	0.154	0.0886	1.74	1.801	0.2794	6.45
	31	6	0.168	0.1007	1.67	2.153	0.3681	5.85
Foreman	40	1	0.0429	0.0816	0.53	0.4401	0.099	4.45
	39	2	0.0439	0.0812	0.54	0.4761	0.1215	3.92
	38	3	0.0609	0.0871	0.69	0.7341	0.1658	4.43
	34	4	0.0973	0.0883	1.10	1.2915	0.2166	5.96
	34	5	0.1191	0.0941	1.27	1.5398	0.3581	4.3
	32	6	0.1571	0.1098	1.43	1.8296	0.4883	3.75
Coastguard	38	1	0.0464	0.0779	0.60	0.468	0.09	5.20
	37	2	0.05	0.0771	0.65	0.5316	0.1012	5.26
	37	3	0.074	0.0808	0.92	0.789	0.1287	6.13
	34	4	0.118	0.0869	1.36	1.368	0.1802	7.59
	33	5	0.163	0.0873	1.87	1.833	0.2775	6.61
	31	6	0.179	0.1070	1.67	2.199	0.4085	5.38

**Table 2:** Average Computational Time of Channel Encoding (T_C_) and Average Number of Bit planes (NBP) for Hall, Foreman, and Coastguard Video Sequences with GOP size 2.

Video Details			T_C_ (s)			NBP		
Name	Qp	Qm	DIS	QUATRID	CTIR_C_	DIS	QUATRID	BPR (%)
Hall	37	1	0.396	0.012	33	40	1.15	97.13
	36	2	0.452	0.025	18.08	44	2.40	94.55
	36	3	0.683	0.0436	15.67	68	4.14	93.91
	33	4	1.252	0.0669	18.72	120	6.74	94.38
	33	5	1.647	0.1909	8.63	144	19.29	86.61
	31	6	1.985	0.280	7.09	180	27.24	84.87
Foreman	40	1	0.397	0.0175	22.69	40	1.73	95.68
	39	2	0.432	0.0401	10.78	44	4.01	90.88
	38	3	0.673	0.0786	8.57	68	7.91	88.37
	34	4	1.194	0.1283	9.31	120	13.08	89.1
	34	5	1.421	0.2641	5.38	144	26.87	81.34
	32	6	1.829	0.3784	4.84	180	38.47	78.63
Coastguard	38	1	0.422	0.0124	34.03	40	1.22	96.95
	37	2	0.4815	0.0240	20.06	44	2.39	94.57
	37	3	0.715	0.048	14.90	68	4.75	93.02
	34	4	1.284	0.0933	13.76	120	8.42	92.98
	33	5	1.67	0.1902	8.78	144	18.75	86.98
	31	6	2.02	0.3015	6.70	180	26.83	85.10

**Table 3:** Rate-Distortion (RD) Performance Analysis of DIS, Intra, and QUATRID codecs for Foreman Video Sequence with GOP size 2.

Video Details			Rate (kbps)			PSNR (dB)		
Name	Qp	Qm	DIS	Intra	QUATRID	DIS	Intra	QUATRID
Foreman	40	1	70.17	71.96	44.22	28.52	27.45	28.26
	39	2	83.89	91.45	61.23	29.23	28.69	28.74
	38	3	94.62	102.58	75.30	29.74	29.33	29.18
	34	4	154.29	142.84	112.18	32.26	31.23	31.15
	34	5	161.59	159.28	147.99	32.31	31.87	31.17
	32	6	210.21	200.24	191.24	33.55	33.33	31.997

**Table 4:** Rate-Distortion (RD) Performance Analysis of DIS, Intra, and QUATRID codecs for Coastguard Video Sequence with GOP size 2.

Video Details			Rate (kbps)			PSNR (dB)		
Name	Qp	Qm	DIS	Intra	QUATRID	DIS	Intra	QUATRID
Coastguard	38	1	79.92	115.32	63.363	28.55	28.11	28.62
	37	2	97.39	130.65	75.31	29.26	28.64	29.21
	37	3	101.44	130.65	80.85	29.31	28.64	29.22
	34	4	154.12	193.05	124.19	31.07	30.52	30.564
	33	5	170.96	222.61	158.95	31.61	31.25	30.999
	31	6	228.66	250.66	190.21	32.87	31.94	31.94

**Table 5:** Rate-Distortion (RD) Performance Analysis of DISCOVER, Intra, and QUATRID codecs for Hall Video Sequence with GOP size 2.

Video Details			Rate (kbps)			PSNR (dB)		
Name	Q_P_	Qm	DIS	Intra	QUATRID	DIS	Intra	QUATRID
Hall	37	1	83.72	144.59	77.16	31.46	31.42	31.52
	36	2	93.33	155.12	87.31	32.03	31.98	31.97
	36	3	96.37	171.32	98.30	32.07	32.71	31.97
	33	4	131.46	206.98	123.81	34.35	34.33	33.93
	33	5	134.43	224.27	144.64	34.36	35.06	33.95
	31	6	168.57	248.6	178.97	35.93	35.94	35.11

**Table 6:** Evaluation of Average Feedback Channel Requests per Frame of DIS and QUATRID.

Video	Average Feedback Channel Requests (per Frame)		Feedback Requests Reduction Ratio $\left(feedback_{DIS}÷feedback_{QUATRID}\right)$
	DIS	QUATRID	
Hall	547.34	192.149	2.85
Foreman	710.64	217.357	3.26
Coastguard	826	205.882	4.02

**Table 7:** Summarize the Performance Comparison of DIS, QUATRID, and Intra codecs.

Video	CTIR_f_	CTIR_C_	BPR (%)	Bjøntegaard Delta Performance (DIS vs. QUATRID)						Bjøntegaard Delta Performance (Intra vs. QUATRID)	
				BD Rate (%) at			BD PSNR (dB) at			BD Rate (%)	BD PSNR (dB)
				RD_Low_	RD_High_	RD_Overall_	RD_Low_	RD_High_	RD_Overall_		
Hall	4.5–9.3	7–33	84–97	−2.7	9.18	4.2	0.08	−0.50	−0.23	−32.45	2.31
Foreman	3.7–5.9	4.8–22.6	78–95	−15.52	21.67	−5.4	0.44	−0.71	0.061	−18.86	0.69
Coastguard	5.2–7.5	8.7–34	86–96	−18.04	9.8	−10.48	0.68	−0.26	0.32	−37.41	1.74

**Table 8:** RD Performance Comparison of Intra and QUATIRD Codecs for Additional Test Sequences.

Video Details			Rate (kbps)		PSNR (dB)		NBP
Name	Qp	Qm	Intra	QUATRID	Intra	QUATRID	QUATRID
Akiyo	38	1	170.83	90.68	32.08	33.41	1.04
	36	2	195.93	106.24	33.31	34.3	1.75
	34	3	226.96	125.09	34.82	35.63	3.03
	33	4	240.24	138.80	35.43	36.11	5.13
	31	5	285.92	175.84	36.81	37.20	10.74
	29	6	320.89	204.56	38.17	38.43	15.08
Salesman	37	1	258.86	135.53	30.26	30.99	1.01
	36	2	278.00	147.70	30.69	31.20	1.54
	34	3	339.69	182.16	32.15	32.52	2.66
	32	4	406.88	222.46	33.37	33.50	4.61
	31	5	455.58	263.44	34.28	34.07	11.20
	31	6	455.58	273.03	34.28	34.27	15.77

**Table 9:** Average Computational Time of Full Wyner-Zive (T_f_), Channel Encoding (T_C_), and Average Number of Bit planes (NBP) for high resolution videos with GOP size 2.

Video Details			T_f_			T_C_			NBP		
Name	Qp	Qm	DIS	QUATRID	CTIR_f_	DIS	QUATRID	CTIR_C_	DIS	QUATRID	BPR (%)
Bus (352 × 288)	37	1	1.76	1.09	1.61	1.51	0.707	2.14	160	71	55.63
	33	4	4.9	3.57	1.37	4.3	2.91	1.48	480	330	31.25
Coastguard (352 × 288)	38	1	1.59	0.93	1.7	1.37	0.59	2.32	160	68	57.5
	34	4	5.05	4.33	1.17	4.43	3.61	1.23	480	403	16.05

## Data Availability

Not applicable.

## References

[1] Slepian D., Wolf J. (1973). Noiseless coding of correlated information sources. IEEE Trans. Inf. Theory.

[2] Wyner A., Ziv J. (1976). The rate-distortion function for source coding with side information at the decoder. IEEE Trans. Inf. Theory.

[3] Ma T., Hempel M., Peng D., Sharif H. (2012). A survey of energy-efficient compression and communication techniques for multimedia in resource constrained systems. IEEE Commun. Surv. Tutor..

[4] Hu C., Zhao Y., Yu L., Jiang Y., Xiong Y. (2020). A simple encoder scheme for distributed residual video coding. Multimed. Tools Appl..

[5] Artigas X., Ascenso J., Dalai M., Klomp S., Kubasov D., Ouaret M. The DISCOVER codec: Architecture, techniques and evaluation. Proceedings of the Picture Coding Symposium (PCS’2007).

[6] Trieu Duong D., Cong H.P., Van X.H. (2019). A novel consistent quality driven for JEM based distributed video coding. Algorithms.

[7] Verbist F., Deligiannis N., Satti S.M., Munteanu A., Schelkens P. (2012). Iterative Wyner-Ziv decoding and successive side-information refinement in feedback channel-free hash-based distributed video coding. Applications of Digital Image Processing XXXV.

[8] Lei T.C.-W., Tseng F.-S. (2017). High performance and low complexity decoding light-weight video coding with motion estimation and mode decision at decoder. EURASIP J. Image Video Process..

[9] Puri R., Majumdar A., Ramchandran K. (2007). PRISM: A video coding paradigm with motion estimation at the decoder. IEEE Trans. Image Process..

[10] Aaron A., Girod B. Wyner-Ziv video coding with low-encoder complexity. Proceedings of the Picture Coding Symposium, PCS 2004.

[11] Aaron A., Rane S., Girod B. Wyner-Ziv video coding with hash-based motion compensation at the receiver. Proceedings of the 2004 International Conference on Image Processing, ICIP’04.

[12] Li Z., Liu L., Delp E.J. (2006). Rate distortion analysis of motion side estimation in Wyner–Ziv video coding. IEEE Trans. Image Process..

[13] Ascenso J., Pereira F. Adaptive hash-based side information exploitation for efficient Wyner-Ziv video coding. Proceedings of the 2007 IEEE International Conference on Image Processing.

[14] Ascenso J., Pereira F. Low complexity intra mode selection for efficient distributed video coding. Proceedings of the 2009 IEEE International Conference on Multimedia and Expo.

[15] Verbist F., Deligiannis N., Satti S.M., Schelkens P., Munteanu A. (2013). Encoder-driven rate control and mode decision for distributed video coding. EURASIP J. Adv. Signal Process..

[16] Brites C., Ascenso J., Pereira F. (2013). Side information creation for efficient Wyner–Ziv video coding: Classifying and reviewing. Signal Process. Image Commun..

[17] Jia Y., Wang Y., Song R., Li J. (2015). Decoder side information generation techniques in Wyner-Ziv video coding: A review. Multimed. Tools Appl..

[18] Deligiannis N., Barbarien J., Jacobs M., Munteanu A., Skodras A., Schelkens P. (2011). Side-information-dependent correlation channel estimation in hash-based distributed video coding. IEEE Trans. Image Process..

[19] Deligiannis N., Munteanu A., Clerckx T., Cornelis J., Schelkens P. (2009). Overlapped block motion estimation and probabilistic compensation with application in distributed video coding. IEEE Signal Process. Lett..

[20] Verbist F., Deligiannis N., Jacobs M., Barbarien J., Schelkens P., Munteanu A., Cornelis J. (2013). Probabilistic motion-compensated prediction in distributed video coding. Multimed. Tools Appl..

[21] Deligiannis N., Verbist F., Iossifides A.C., Slowack J., Van de Walle R., Schelkens P., Munteanu A. (2012). Wyner-Ziv video coding for wireless lightweight multimedia applications. EURASIP J. Wirel. Commun. Netw..

[22] Chien W.-J., Karam L.J. (2009). Transform-domain distributed video coding with rate–distortion-based adaptive quantisation. IET Image Process..

[23] Wu B., Zhang N., Ma S., Zhao D., Gao W. (2014). Optimal entropy-constrained non-uniform scalar quantizer design for low bit-rate pixel domain DVC. Multimed. Tools Appl..

[24] Zhang L., Peng Q., Wu X. (2017). Perception-based adaptive quantization for transform-domain Wyner-Ziv video coding. Multimed. Tools Appl..

[25] Sofke S., Pereira F., Müller E. (2009). Dynamic quality control for transform domain Wyner-Ziv video coding. EURASIP J. Image Video Process..

[26] Abou-Elailah A., Dufaux F., Farah J., Cagnazzo M., Pesquet-Popescu B. (2012). Fusion of global and local motion estimation for distributed video coding. IEEE Trans. Circuits Syst. Video Technol..

[27] Li Y., Zhao D., Ma S., Gao W. (2009). Distributed video coding based on the human visual system. IEEE Signal Process. Lett..

[28] Bai H., Wang A., Zhao Y., Pan J.-S., Abraham A. (2011). Distributed Multiple Description Coding: Principles, Algorithms and Systems.

[29] Aaron A., Rane S.D., Setton E., Girod B. (2004). Transform-domain Wyner-Ziv codec for video. Visual Communications and Image Processing 2004.

[30] Aaron A., Zhang R., Girod B. Wyner-Ziv coding of motion video. Proceedings of the Conference Record of the Thirty-Sixth Asilomar Conference on Signals, Systems and Computers.

[31] Aaron A., Varodayan D., Girod B. Wyner-Ziv residual coding of video. Proceedings of the Picture Coding Symposium, PCS-2006.

[32] HU C.-Y., HU B.-J. (2016). Encoder rate control algorithm based on scrambling with pseudo-random code for distributed residual coding of video. Acta Electonica Sin..

[33] Hu C., Hu B., Tu W., Xiong Y. (2018). A low-complexity and efficient encoder rate control solution for distributed residual video coding. Multimed. Tools Appl..

[34] Said A., Pearlman W.A. (1996). A new, fast, and efficient image codec based on set partitioning in hierarchical trees. IEEE Trans. Circuits Syst. Video Technol..

[35] Badem M., Arachchi H.K., Worrall S., Kondoz A. Transform domain residual coding technique for distributed video coding. Proceedings of the Picture Coding Symposium (PCS’2007).

